# Remnant Pancreas Volume Affects New-Onset Impaired Glucose Homeostasis Secondary to Pancreatic Cancer

**DOI:** 10.3390/biomedicines12081653

**Published:** 2024-07-24

**Authors:** Jie Yang, Chunlu Tan, Ya Liu, Zhenjiang Zheng, Xubao Liu, Yonghua Chen

**Affiliations:** 1 Division of Pancreatic Surgery, Department of General Surgery, West China Hospital of Sichuan University, Chengdu 610041, China; 2 Department of Biotherapy, State Key Laboratory of Biotherapy and Cancer Center, West China Hospital of Sichuan University, Chengdu 610065, China; 3 Department of Thyroid and Breast Surgery, Chengdu Second People’s Hospital, Chengdu 610041, China

**Keywords:** pancreatic ductal adenocarcinoma, diabetes mellitus, beta-cell function, remnant pancreas volume, tumor stage

## Abstract

Background: New-onset diabetes (NOD) has been identified as a high-risk factor for the early detection of pancreatic ductal adenocarcinoma (PDAC). The role of tumor volume and remnant pancreas volume (RPV) in the progression from normal to NOD in PDAC patients is not fully illustrated yet. Methods: In this cross-sectional study, glycemic metabolism traits of 95 PDAC patients before pancreatic surgery were described and compared with chronic pancreatitis and type 2 diabetes mellitus patients based on the oral glucose tolerance test. The remnant RPV and tumor volume, calculated by three-dimensional reconstruction of radiological images, were included in the ordinal logistic regression models. Results: The prevalence of NOD was high among PDAC patients (38.9%). However, normal glucose tolerance (NGT) or prediabetes mellitus status were present as more than half (24/44) of advanced tumor stage patients. Indexes reflecting beta-cell function but not insulin sensitivity gradually worsened from NGT to NOD patients (all *p* < 0.05). The remnant pancreas volume (RPV) was identified as a potential protective factor for diabetes secondary to PDAC (odds ratio 0.95, 95% CI [0.92, 0.97], *p* < 0.001). Conclusions: Reduced RPV causing beta-cell dysfunction might be one of the mechanisms of NOD secondary to PDAC. Subjects with sufficient pancreas volume could not be detected earlier when regarding patients with NOD as the population at risk for PDAC.

## 1. Introduction

Pancreatic ductal adenocarcinoma (PDAC) carries a dismal prognosis, as around 85% of patients are unresectable at diagnosis [1,2]. Thus, early detection is likely to significantly improve overall survival. New-onset diabetes (NOD) has been identified as one of the high-risk factors for the early detection of PDAC [3]. Indeed, the association between diabetes mellitus (DM) and PDAC has been known for over a century. Subjects with NOD have a 6- to 8-fold higher risk of developing PDAC compared to the general population [4]. Furthermore, the incidence of cancer development related NOD in PDAC patients is much higher than other malignancies, despite PDAC sharing canonical risk factors with diabetes such as age and obesity [5,6].

The process from normal glucose tolerance (NGT) to prediabetes mellitus (preDM) and then NOD in PDAC patients is associated with increasing tumor volume [7]. However, in existing studies, the prevalence of new-onset impaired glucose homeostasis (including preDM and NOD) secondary to PDAC has a huge range from 27% to 75%, despite being based on the ascertainment method of glucose tolerance status [8,9,10,11]. Moreover, NOD secondary to PDAC could occur before the visible appearance of the tumor in the pancreas by CT scan, whereas it might not present in some patients with advanced tumor stage [10,12]. The latter could be neglected in the early detection of PDAC in the context of NOD.

The impaired β-cell function has been shown to be the underlying mechanism in diabetes secondary to PDAC [8,13,14]. One hypothesis is that the local effects of tumor infiltration and pancreatic ductal obstruction could result in the loss of pancreas volume and then insulin deficiency [15]. Recent studies provide insight into the correlation between pancreas volume and DM. Pancreas volume is recognized as a novel noninvasive biomarker for predicting the progression of type 1 DM [16,17]. Related findings have also been reported in a Mendelian randomization study that provides evidence for a causal effect of pancreas volume in the decreased risk of type 2 DM (T2DM) [18]. Whether Pancreas volume plays a critical role in NOD secondary to PDAC is unknown.

In the present study, the epidemiological characteristics of glucose tolerance status in patients with PDAC have been investigated by oral glucose tolerance test (OGTT) prior to pancreatic surgery. Furthermore, glycemic metabolism traits of PDAC were described by demonstrating the changes of OGTT-based curves and glucose metabolic index in subjects with different glucose tolerance statuses and the comparison with chronic pancreatitis (CP) and T2DM patients. In addition, a novel indicator of new-onset impaired glucose homeostasis secondary to PDAC were provided based on the remnant pancreas volume (RPV) calculated by three-dimensional (3D) reconstruction of CT images to explain why some PDAC patients might not develop NOD until an advanced stage.

## 2. Materials and Methods

### 2.1. Study Design

**Figure 1:**
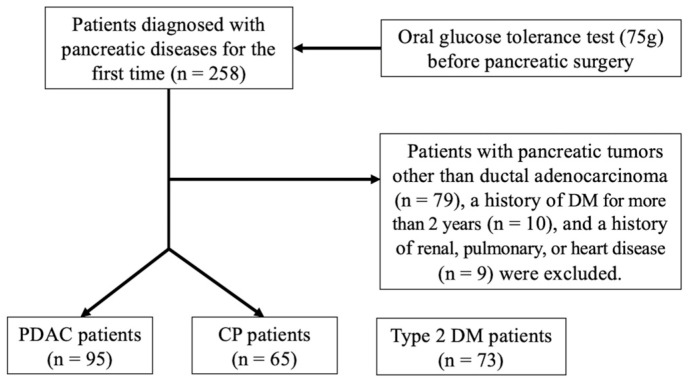
Flow chart of the study.

In this cross-sectional study, from January 2017 to December 2019, 258 patients with clinically diagnosed pancreatic diseases for the first time were invited to participate in a screen for abnormalities in glucose metabolism by OGTT before pancreatic surgery. The clinical diagnosis was confirmed by pathological analysis after surgery or endoscopic ultrasonography. The initial screening included medical history and physical examination of all participating subjects. Weight and height were measured before the OGTT, and body mass index (BMI) was subsequently calculated. Exclusion criteria included patients with pancreatic tumors other than ductal adenocarcinoma, a history of renal, pulmonary, heart disease, gastric bypass surgery, type 1 DM, or DM for more than 2 years. As shown in Figure 1, 95 patients with PDAC (35 females, 60 males; mean age, 59.8 ± 11.6 years) and 65 patients with CP were involved. The tumor stage of PDAC patients was classified according to the 8th Edition American Joint Committee on Cancer staging system [19]. A total of 73 patients without pancreatic disease who were diagnosed with T2DM in the endocrinology department of our hospital during the same period were also included in further analysis. These T2DM patients were not treated with thiazolidinediones, incretin mimetics, or dipeptidyl peptidase 4 inhibitors.

### 2.2. Biochemical Parameters

A laboratory profile (hemoglobin, leukocytes, transaminases, total bilirubin, total bile acids, triglycerides, g-glutamyl transpeptidase, creatinine, estimated glomerular filtration rate [eGFR], CA19-9, and HbA1c) was performed in all subjects. After an overnight fast of at least 10 h, a 75 g OGTT was implemented at 8:30 a.m., with blood samples drawn at 0, 30, 60, 120, and 180 min following glucose ingestion for the measurement of glucose, insulin, and C-peptide concentrations. Glucose tolerance status was determined on the OGTT according to the American Diabetes Association recommendations criteria for preDM and diabetes [20]. When fasting plasma glucose ≥6.1 mmol/L and <7.0 mmol/L, or 2 h plasma glucose ≥7.8 mmol/L and <11.1 mmol/L, preDM was diagnosed. DM was diagnosed if fasting plasma glucose ≥7.0 mmol/L or 2 h plasma glucose ≥11.1 mmol/L. NGT was diagnosed if fasting plasma glucose was <6.1 mmol/L and 2 h plasma glucose <7.8 mmol/L. NOD secondary to PDAC was defined as diabetes less than 2 years in duration [10].

For the homeostatic model assessment (HOMA), beta cell function (HOMA2-%beta), insulin resistance (HOMA2-IR), and insulin sensitivity (HOMA2-S) were calculated using the HOMA2 Calculator (version 2.2.3) [21]. For the glucose metabolic indexes based on the OGTT test, the area under the curve (AUC) for glucose, insulin, and C-peptide was calculated using the trapezoidal rule. The insulinogenic index (IGI) was calculated as the ratio of the increment of plasma insulin to the increment in glucose during the first 30 min of OGTT to measure the first-phase insulin secretion and beta-cell function [22]. The Matsuda index was adopted as a measure of insulin sensitivity [23]. To evaluate β-cell function corrected for the degree of insulin sensitivity, insulin secretion–sensitivity index-2 (ISSI-2, or disposition index) was calculated as the product of (i) insulin secretion measured by the ratio of the total area-under-the-insulin curve (AUCinsulin) to the area-under-the-glucose curve (AUCglucose) and (ii) insulin sensitivity measured by the Matsuda index [24]. Hepatic insulin clearance (HIC) was calculated as the ratio of fasting C-peptide and fasting plasm insulin, and 3 h postprandial HIC was calculated from the ratio of C-peptide AUC0–120 min and plasma insulinAUC0–180 min [25].

### 2.3. Calculation of Remnant Pancreas Volume

We used the Medical Visualization Workstation to construct 3D images by integrating multidetector CT (MDCT) images (Version 1). This software offers a standardized analysis of organ or tumor anatomy and volume based on two-dimensional MDCT images. Serial transverse enhanced MDCT scans were performed before the OGTT test. CT images were first preprocessed by the Gaussian smoothing algorithm. Then, the organ or tumor was segmented by using spherical region growing, median smoothing, and connectivity-based threshold algorithm methods and repaired by freehand tracing. Model reconstruction was carried out by the MarchingCubes 3D reconstruction algorithm, and the reconstructed 3D model was processed by the multilateral contour cutting tool and other model optimization tools. Finally, the volume of the target organ or tumor was calculated by quantitative model analysis tools such as the 3D space length measurement tool and angle measurement tool. The actual RPV was determined by extracting tumor volume and the volume of the extended pancreatic duct, vascular structures, and bile duct. The volumetric analysis was conducted by two senior physicians who were blinded to the outcomes. Tumor location and the diameter of the main pancreatic duct in the neck and tail of the pancreas were also recorded based on the CT images and the intraoperative findings.

### 2.4. Statistical Analysis

Data are presented as frequencies for categorical variables and analyzed by Pearson’s chi-square test or Fisher’s exact test. Continuous variables were expressed as mean ± standard deviation (SD) and were analyzed using the Student *t*-test or Mann–Whitney U nonparametric test. The ordinal logistic regression models were used to compute the odds ratio (OR) with a 95% confidence interval (CI) estimate of preDM and NOD versus NGT. Relationships between RPV and other variables were estimated by linear regression analysis and Pearson correlation. A two-sided *p* value less than 0.05 was regarded as statistically significant (*, *p* < 0.05, **, *p* < 0.01, ***, *p* < 0.001, ****, *p* < 0.0001). All the data were analyzed by SPSS version 24.0 (IBM, New York, NY, USA) and GraphPad Prism version 8.2.0 (GraphPad Software, San Diego, CA, USA).

## 3. Results

### 3.1. Glycemic Traits of PDAC Patients

The clinical and metabolic characteristics of included PDAC patients are summarized in Table 1. The prevalence of NGT, preDM, and NOD in those PDAC patients was 17.9%, 43.2%, and 38.9%. However, 54.5% (24/44) of patients with advanced tumor stage (III or IV) presented as only NGT or preDM but not NOD. For early-stage (I or II) patients, only 33.3% (17/51) could be diagnosed with DM. The characteristics and glucose tolerance status of involved CP patients were listed in Table S1.

### 3.2. Glucose Metabolism Traits of PDAC Patients with Different Glucose Tolerance Statuses

To investigate the glycemic traits of PDAC patients, we demonstrated the changes of OGTT-based curves and glucose metabolic index in PDAC patients with different glucose tolerance statuses and compared them with CP and T2DM patients. As plasma glucose levels increased in PDAC patients (Figure S1A–C), the NOD group showed a simultaneous decrease in insulin and C-peptide levels compared with the preDM group (Figure S1D–I). This difference was not found in the comparison of the NGT and preDM groups. The same trend was observed in CP patients. However, when compared with T2DM patients, plasma insulin and C-peptide levels at each point of the OGTT curve from 0 to 120 min were significantly lower in NOD secondary to PDAC (all *p* < 0.05, Figure S1F,I).

**Figure 2:**
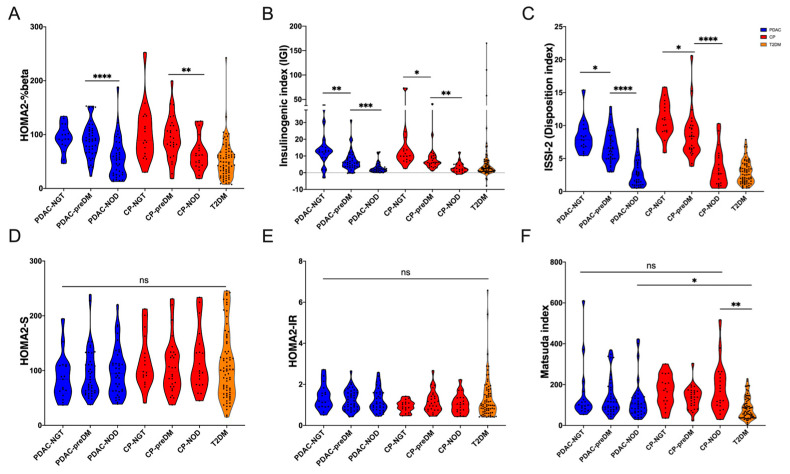
Glucose metabolism indexes in PDAC and CP patients with different glucose tolerance statuses (NGT, preDM, and NOD) and type 2 DM patients. (**A**) HOMA2-%beta, (**B**) insulinogenic index, (**C**) insulin secretion–sensitivity index-2 (ISSI-2, or disposition index), (**D**) HOMA2-S, (**E**) HOMA2-IR, (**F**) Matsuda index. * *p* value less than 0.05, ** *p* value less than 0.01, *** *p* value less than 0.001, **** *p* value less than 0.0001; ns, no significance.

For the glucose metabolism indexes reflecting beta-cell function, the decrease of the HOMA2-%beta index was also found in the comparison between preDM and NOD patients (*p* < 0.0001, Figure 2A). With the decrease of glucose tolerance in PDAC patients (from NGT to preDM and the NOD), IGI and ISSI-2 were also gradually decreased (all *p* < 0.05, Figure 2B,C). These tendencies were also similar to CP patients with different glucose tolerance statuses. Homeostatic model assessment of insulin resistance, insulin sensitivity (HOMA2-IR and HOMA2-S), and Matsuda index showed no statistical difference between the three groups of PDAC patients (all *p* > 0.05, Figure 2D–F), whereas the Matsuda index of T2DM patients was lower than that of NOD secondary to PDAC patients (*p* < 0.05). This revealed that insulin resistance was not changed among PDAC patients with different glucose tolerance statuses. Combined with the difference in HIC and 3 h postprandial HIC that were also not observed (Figure S2A,B), the gradual decline of beta-cell function could be one of the main reasons for the eventual development of NOD in PDAC patients.

### 3.3. Risk Factors of preDM and NOD Secondary to PDAC

**Figure 3:**
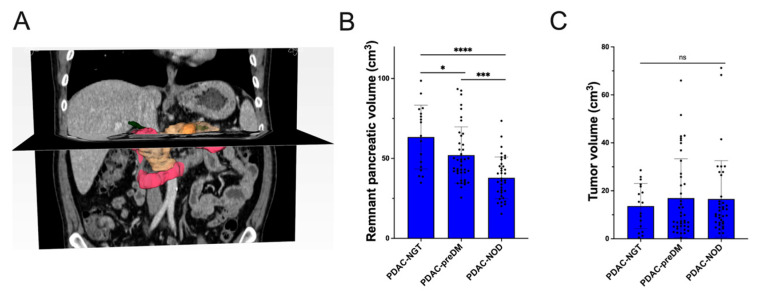
Calculation of tumor volume and remnant pancreas volume based on three-dimensional reconstruction of CT images. (**A**) Three-dimensional image of the pancreas and surrounding organs. The yellow region represents the pancreas, the orange region represents the tumor, the red region represents the duodenum, and the green region represents the bile and pancreatic ducts. The remnant pancreas volume was determined by identifying the entire pancreatic region and subtracting the tumor volume and the volume of the extended pancreatic duct and bile duct. Comparison of (**B**) remnant pancreas volume and (**C**) tumor volume between PDAC patients with different glucose tolerance statuses. * *p* value less than 0.05, *** *p* value less than 0.001, **** *p* value less than 0.0001; ns, no significance.

To investigate the risk factor for the decline of glucose tolerance in PDAC patients, tumor volume and RPV were calculated and involved with further analysis (Figure 3A). RPV gradually declined in patients with NGT (63.38 ± 19.96 cm^3^), preDM (52.07 ± 17.67 cm^3^), and NOD (37.87 ± 13.02 cm^3^, all *p* < 0.05, Figure 3B and Table S2). Tumor volume showed no statistical difference between PDAC patients with different glucose tolerance statuses (NGT [13.61 ± 9.39 cm^3^], preDM [16.95 ± 16.41 cm^3^], and NOD [16.58 ± 15.94 cm^3^], all *P* > 0.05, Figure 3C and Table S2). The ordinal logistic regression analysis showed that for each additional cubic centimeter of RPV, the proportional odds of having impaired glucose homeostasis (NOD versus preDM, preDM versus NGT) were 5% lower (odds ratio 0.95, 95% CI [0.92, 0.97], *p* < 0.001, Table 2). Patients with advanced tumor stage (IIB~IV) were more likely to develop preDM and NOD (odds ratio 2.65, 95% CI [1.05, 6.23], *p* = 0.039). In addition, for patients with BMI lower than 18.5 or more than 23.9 kg/m^2^, the proportional odds of having impaired glucose homeostasis were 2.54 times higher (odds ratio 2.54, 95% CI [1.03, 6.23], *p* = 0.042).

### 3.4. Correlation between RPV and Glucose Metabolism Indexes

**Figure 4:**
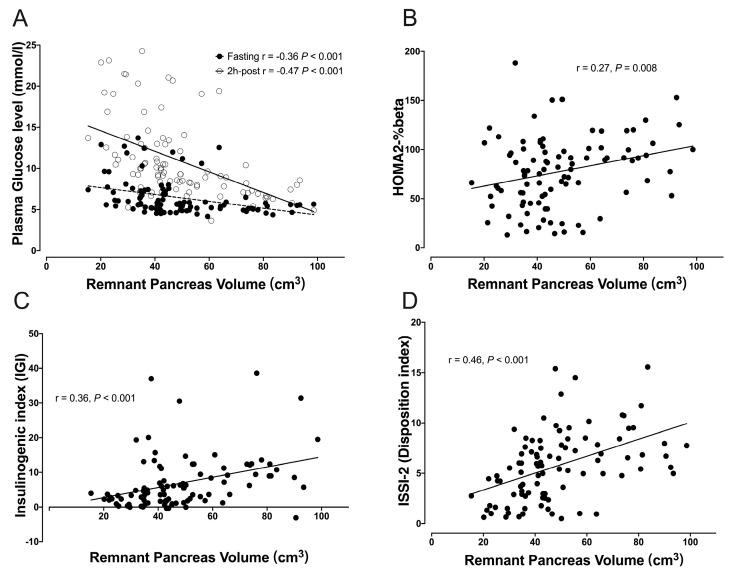
Correlation between remnant pancreas volume and glucose metabolism indexes in PDAC patients. (**A**) Plasma fasting and 2 h glucose levels following OGTT, (**B**) HOMA2-%beta, (**C**) insulinogenic index, and (**D**) ISSI-2.

To further verify the association between RPV and impaired glucose homeostasis in PDAC patients, linear regression analysis was used to assess the relationship between RPV and plasma glucose levels and glucose metabolism indexes reflecting the beta-cell function. The analysis of all PDAC patients revealed strong inverse correlations between RPV and both fasting glucose levels (r = −0.36, *p* < 0.001) and 2 h glucose levels following OGTT (r = −0.47, *p* < 0.001, Figure 4A). Positive correlations were found between RPV and HOMA2-%beta (r = 0.27, *p* = 0.008, Figure 4B), IGI (r = 0.36, *p* < 0.001, Figure 4C), and ISSI-2 (r = 0.46, *p* < 0.001, Figure 4D). No linear correlations were found between PRPV and age, BMI, and tumor volume (all *p* > 0.05, Figure S3).

## 4. Discussion

Our findings suggest that the prevalence of new-onset impaired glucose homeostasis is high in PDAC patients (82.1%). However, more than half of patients with advanced tumor stage (III or IV) present as only NGT or preDM but not NOD. To figure out the mechanism underlying NOD secondary to PDAC, in comparison to CP, whose deficient insulin production is caused by beta-cell destruction [26], the overall trend in the change of the OGTT-based curves and beta-cell function indexes (HOMA2-%beta, IGI, ISSI-2) was consistent between these two most commonly identified causes of type 3c DM. Moreover, there was no difference in insulin resistance and sensitivity index (HOMA2-IR, MHOMA2-S, Matsuda index) between PDAC patients with different glucose tolerance statuses, and NOD patients secondary to PDAC have significantly lower insulin resistance levels than T2DM patients. Thus, beta-cell dysfunction might be the primary reason for the new-onset impaired glucose homeostasis in PDAC patients. Ordinal logistic regression analysis showed that BMI, tumor stage, and RPV, but not tumor volume, were associated with the severity of impaired glucose homeostasis. The strong inverse correlation between RPV and plasma glucose levels and the positive correlation with beta-cell function indexes could further verify this relevance.

**Figure 5:**
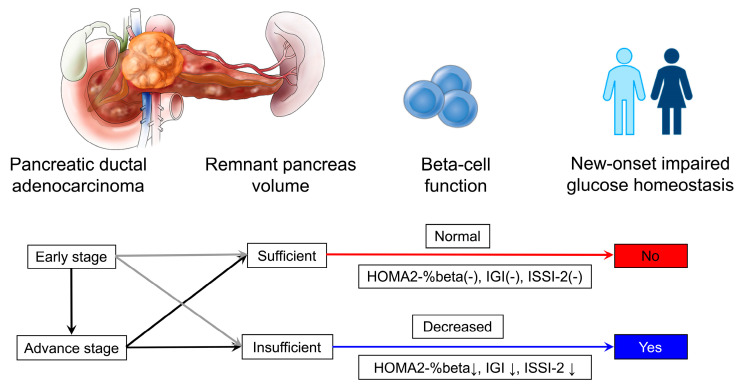
Graphic of the main findings of the study. Given the protective efficacy of RPV, when regarding patients with NOD as the risk population of PDAC, only early-stage patients with insufficient RPV could be identified due to the decreased beta-cell function. While early-stage patients with sufficient RPV might not develop NOD, when the PDAC progresses to a certain degree (such as the advanced stage) and causes insufficient RPV, subjects could be detected through NOD.

The duration from NOD to the diagnosis of PDAC has been well elaborated. A population-based epidemiologic study showed that impaired fasting glucose levels were seen 36 to 30 months prior to the diagnosis of PDAC [7]. Data from a high-volume pancreatic disease center also revealed that the median duration of NOD before PDAC diagnosis was 0.5 to 35 months (20 of 24 patients with tumor stage IIb to IV) [11]. Despite the specific period from the onset of PDAC to the development of NOD being hard to figure out, the RPV could disturb this time course. Given the potential protective efficacy of RPV, when regarding patients with NOD as the risk population of PDAC, only early-stage patients with insufficient RPV could be identified, while early-stage patients with sufficient RPV might not develop NOD (Figure 5). When the PDAC progresses to a certain degree (such as the advanced stage) and causes insufficient RPV, subjects could be detected through NOD. However, some patients with advanced tumor stage who still have sufficient pancreas volume only present as NGT or preDM but not NOD (more than half of the patients in the present study). This weakens the value of NOD in the early diagnosis of PDAC.

Although no correlation was found between RPV and age in the context of PDAC, previous work has shown that pancreas volume reaches a plateau at the age of around 50 years and then declines [27]. That might explain why NOD is currently most prevalent in the elderly (≥50 years) population [3]. Abnormal BMI (<18.5, >23.9 kg/m^2^) also showed an increased risk of having impaired glucose homeostasis, since pancreatic fat increased proportionately with obesity, and the pancreatic parenchyma volume decreased, resulting in a proportionately higher fat content. Similar findings have been reported that individuals with diabetes after acute pancreatitis have a significantly higher pancreatic fat infiltration [28]. In a recent prospective pilot study involving patients with newly elevated glycemic parameters to detect PDAC, 5 of 93 patients are identified with pancreatic fat infiltration [29]. On the other hand, studies have discussed the relationship between pancreatic cancer associated diabetes mellitus and cachexia. The relatively high prevalence of cachexia and percentage of weight loss were also related to patients with diabetes mellitus secondary to PDAC [30]. This was consistent with our findings that low BMI (<18.5 kg/m^2^) was also recognized as a risk factor of preDM and NOD in PDAC patients. The complex relationship between pancreatic volume, BMI, and age also increases the difficulty of clarifying the progression of NOD secondary to PDAC.

A confusing relationship exists between tumor volume and new-onset impaired glucose homeostasis. In a comprehensive study by Sharma and colleagues, fasting plasma glucose levels increased with tumor volume [7]. However, early insights into tumor volume and glucose metabolism showed that impaired glucose tolerance or DM in PDAC patients occurs well before the visible appearance of the tumor in the pancreas [12,31]. In the present cohort, tumor volume did not differ between PDAC patients with NGT, preDM, or NOD. We are also deep into the relationship between tumor location, dilation of the main pancreatic duct, and new-onset impaired glucose homeostasis. Although islet distribution is over 2-fold higher in the pancreatic tail than in the head and body [32], and pancreatic head and neck tumors could obstruct the main pancreatic duct and then lead to atrophy of the distal pancreas, no significant manifestation was found.

Besides RPV, the intrinsic tumor factors could lead to impaired glucose homeostasis in PDAC patients. The greater effect of the advanced tumor stages on glucose metabolism might reflect the progression of the intrinsic tumor factors. For instance, higher lymph node involvement was also found in PDAC patients with higher fasting plasma glucose levels [7]. There is also evidence that the resection of tumors and effective chemotherapy could improve glucose metabolic defects during the treatment of PDAC patients [7,10,33]. Indeed, studies have worked to find that adrenomedullin and exosomes derived from PDAC cause paraneoplastic dysfunction of human beta-cells and suppress insulin secretion thereby causing hyperglycemia [34,35,36]. Further investigations are needed to evaluate the RPV combined with biomarkers to reflect the degree of impact on beta-cell function in PDAC patients. Furthermore, the changes in the gut microbiome of PDAC patients may also have a potential impact on beta-cell function [37]. In addition, the combined use of multiple predictive methods, such as polygenic risk scores, can more accurately predict PDAC in individuals with NOD [38].

For the limitations of the study, the specific period from the onset of PDAC to the development of NOD secondary to PDAC could not be determined. Furthermore, the hyperglycemic clamp test was not used to further validate the beta-cell function. A multicenter study with a larger sample size is needed to confirm this result.

## 5. Conclusions

Our study shows that one of the critical mechanisms of NOD secondary to PDAC is the reduced RPV and consequent inadequate insulin secretion but not insulin resistance. Subjects with sufficient pancreas volume could not be detected earlier when regarding patients with NOD as the population at risk for PDAC.

## Figures and Tables

**Table 1:** Characteristics of PDAC patients with different glucose tolerance status.

Characteristics	All Patients (n = 95)	NGT (n = 17)	preDM (n = 41)	NOD (n = 37)	*p* Value 1	*p* Value 2	*p* Value 3
Sex (male)	60	7	30	23	0.035	0.338	0.238
Age (year)	59.8 ± 11.6	59.3 ± 11.5	56.9 ± 13.3	63.2 ± 8.6	0.519	0.014	0.169
BMI (kg/m^2^)	22.17 ± 2.97	22.84 ± 2.99	21.82 ± 2.64	22.28 ± 3.35	0.220	0.498	0.587
Smoking history	36	4	17	15	0.241	0.255	0.061
Drinking history	34	6	15	13	>0.999	>0.999	>0.999
Tumor location					0.765	0.815	>0.999
Head and neck	60	10	27	23			
Body and tail	35	7	14	14			
Diameter of the main pancreatic duct							
In the pancreatic neck (mm)	4.7 ± 3.8	5.1 ± 4.3	4.1 ± 3.5	5.1 ± 3.9	0.388	0.267	0.998
In the pancreatic tail (mm)	3.0 ± 2.2	2.4 ± 1.3	2.8 ± 2.3	3.6 ± 2.4	0.458	0.146	0.016
Tumor poorly differentiated	33	7	11	15	0.354	0.235	>0.999
Tumor stage					0.022	0.824	0.017
I~II	51	14	20	17			
III~IV	44	3	21	20			
CA19-9 (U/mL)	385.8 ± 395.7	380.5 ± 397.3	361.5 ± 392.8	415.2 ± 407.0	0.868	0.555	0.771
Total bilirubin (μmol/L)	60.5 ± 95.2	52.5 ± 95.3	66.0 ± 96.3	57.9 ± 96.2	0.626	0.712	0.846
Total bile acids (μmol/L)	37.1 ± 76.5	9.3 ± 19.8	5.7 ± 5.2	5.7 ± 5.4	0.279	0.674	0.824
Creatine (μmol/L)	65.2 ± 13.3	62.7 ± 11.5	68.2 ± 14.6	62.9 ± 12.0	0.164	0.083	0.944
eGFR (mL/min/1.73m^2^)	96.8 ± 12.7	97.5 ± 10.8	97.4 ± 15.4	95.9 ± 10.3	0.970	0.622	0.602

PDAC, pancreatic ductal adenocarcinoma; NGT, normal glucose tolerance; NOD, new-onset diabetes; preDM, prediabetes mellitus; BMI, body mass index; eGFR, estimated glomerular filtration rate; *p* value 1, NGT vs. preDM; *p* value 2, preDM vs. NOD; *p* value 3, NGT vs. NOD.

**Table 2:** Ordinal logistic regression for risk factors to preDM and NOD in PDAC patients.

Variables	PDAC	
	*p* Value	OR (95%CI)
Sex (male)	0.627	1.24 (0.52, 2.95)
Age (≥65 years)	0.550	1.32 (0.53, 3.33)
BMI (<18.5, >23.9 kg/m^2^)	0.042	2.54 (1.03, 6.23)
Remnant pancreas volume (cm^3^)	<0.001	0.95 (0.92, 0.97)
Tumor volume (cm^3^)	0.231	1.03 (0.98, 1.09)
Tumor location (head and neck)	0.895	1.07 (0.40, 2.83)
MPD dilation	0.928	1.05 (0.39, 2.84)
Tumor well-differentiated	0.804	0.89 (0.37, 2.17)
Tumor stage (IIB~IV)	0.039	2.56 (1.05, 6.23)

PDAC, pancreatic ductal adenocarcinoma; OR, odds ratio; CI, confidence interval; BMI, body mass index; MPD, main pancreatic duct; preDM, prediabetes mellitus; NOD, new-onset diabetes.

## Data Availability

The original contributions presented in the study are included in the article/Supplementary Material; further inquiries can be directed to the corresponding authors.

## Supplementary Materials

The following supporting information can be downloaded at: https://www.mdpi.com/article/10.3390/biomedicines12081653/s1, Figure S1: (A–C) Plasma glucose levels, (D–F) insulin levels, and (G–I) C-peptide levels based on OGTT curve in PDAC and CP patients with different glucose tolerance statuses (NGT, preDM, and NOD) and type 2 DM patients; Figure S2: (A) Hepatic insulin clearance (HIC) and (B) 3-h postprandial HIC in PDAC and CP patients with different glucose tolerance statuses (NGT, preDM, and NOD) and type 2 DM patients; Figure S3: Correlation between remnant pancreas volume and (A) age, (B) BMI, and (C) tumor volume in PDAC patients; Table S1: Characteristics of CP patients with different glucose tolerance status; Table S2: Remnant pancreas volume and tumor volume based on three-dimensional reconstruction of CT images.
