# Supplementary material for: Remnant Pancreas Volume Affects New-Onset Impaired Glucose Homeostasis Secondary to Pancreatic Cancer
Source: Biomedicines. 2024 Jul 24;12(8):1653. doi: 10.3390/biomedicines12081653 (PMC11351567; doi:10.3390/biomedicines12081653)
Supplement: Supplementary file 1 [file biomedicines-12-01653-s001.zip › biomedicines-3092996-supplementary.pdf]

Supplementary materials

Table S1 Characteristics of CP patients with different glucose tolerance status

Characteristics	All patients (n = 65)	NGT (n = 17)	preDM (n = 28)	NOD (n = 20)	*P* value 1	*P* value 2	*P* value 3
Sex (female)	15	4	8	3	> 0.999	0.319	0.680
Age (year)	49.7 ± 12.4	44.4 ± 13.9	50.4 ± 8.8	53.2 ± 14.3	0.122	0.443	0.066
Body mass index (kg/m^2)	21.90 ± 3.24	21.80 ± 2.53	22.19 ± 3.43	21.58 ± 3.59	0.684	0.553	0.835
Smoke	31	7	11	13	> 0.999	0.143	0.194
Alcohol	27	7	9	11	0.749	0.144	0.515
CA19-9 (U/ml)	27.0 ± 41.1	20.9 ± 30.6	18.6 ± 24.5	43.9 ± 60.0	0.790	0.087	0.160
Total bilirubin (μmol/L)	28.3 ± 75.3	54.6 ± 137.3	15.2 ± 14.7	26.2 ± 54.4	0.286	0.414	0.461
Total bile acids (μmol/L)	14.9 ± 32.4	24.5 ± 57.0	7.0 ± 5.3	18.7 ± 27.1	0.279	0.674	0.824
Creatine (μmol/L)	64.1 ± 15.2	70.1 ± 17.1	62.1 ± 13.9	62.2 ± 14.9	0.106	0.990	0.162
eGFR (ml/min/1.73m^2)	107.2 ± 15.0	106.1 ± 17.9	107.3 ±10.9	108.1 ± 18.3	0.815	0.872	0.756

CP, chronic pancreatitis, NGT, normal glucose tolerance, preDM, prediabetes mellitus, NOD, new-onset diabetes, eGFR, estimated glomerular filtration rate.

Table S2 Remnant pancreas volume and tumor volume based on three-dimensional reconstruction of CT images.

Characteristics	All patients	NGT	preDM	NOD	*P* value 1	*P* value 2	*P* value 3
PDAC							
Tumor Volume (cm^3)	16.21 ± 15.12	13.61 ± 9.39	16.95 ± 16.41	16.58 ± 15.94	0.336	0.921	0.397
RPV (cm^3)	48.56 ± 18.86	63.38 ± 19.96	52.07 ± 17.67	37.87 ± 13.02	0.037	< 0.001	< 0.001
Chronic pancreatitis							
Pancreas volume (cm^3)	45.08 ± 19.48	59.57 ± 17.33	43.40 ± 20.28	34.12 ± 10.46	0.009	0.045	< 0.001

PDAC, pancreatic ductal adenocarcinoma, RPV, Remnant pancreas volume, NGT, normal glucose tolerance, preDM, prediabetes mellitus, NOD, new-onset diabetes

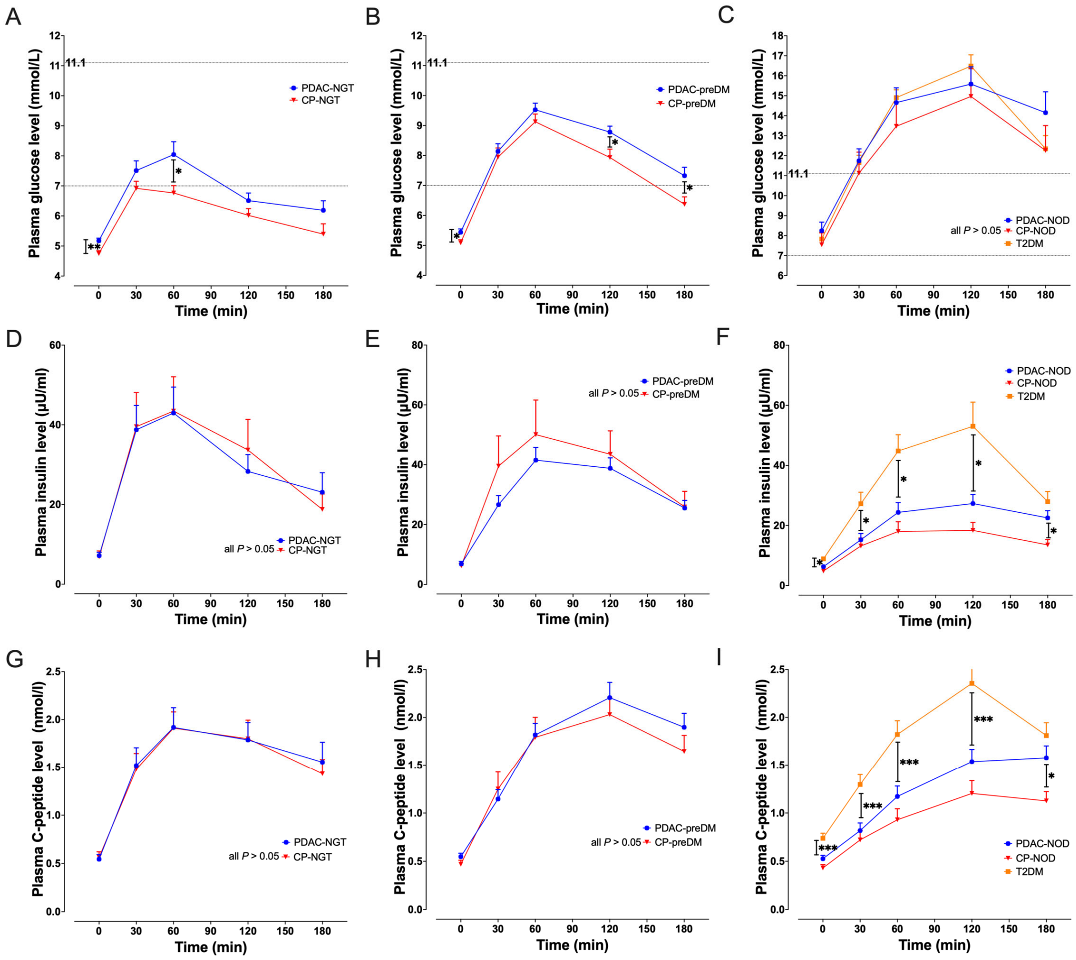

Figure S1 (A, B, C) Plasma glucose levels, (D, E, F) insulin levels, and (G, H, I) C-peptide levels based on OGTT curve in PDAC and CP patients with different glucose tolerance statuses (NGT, preDM, and NOD) and type 2 DM patients.

**P* less than .05, ****P* less than .001

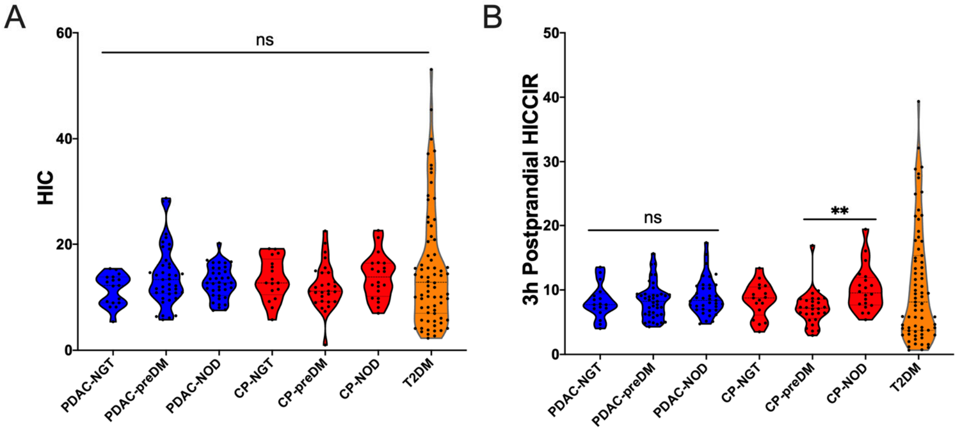

Figure S2 (A) Hepatic insulin clearance (HIC) and (B) 3-hour postprandial HIC in PDAC and CP patients with different glucose tolerance statuses (NGT, preDM, and NOD) and type 2 DM patients.

***P* less than .01, ns, no significance.

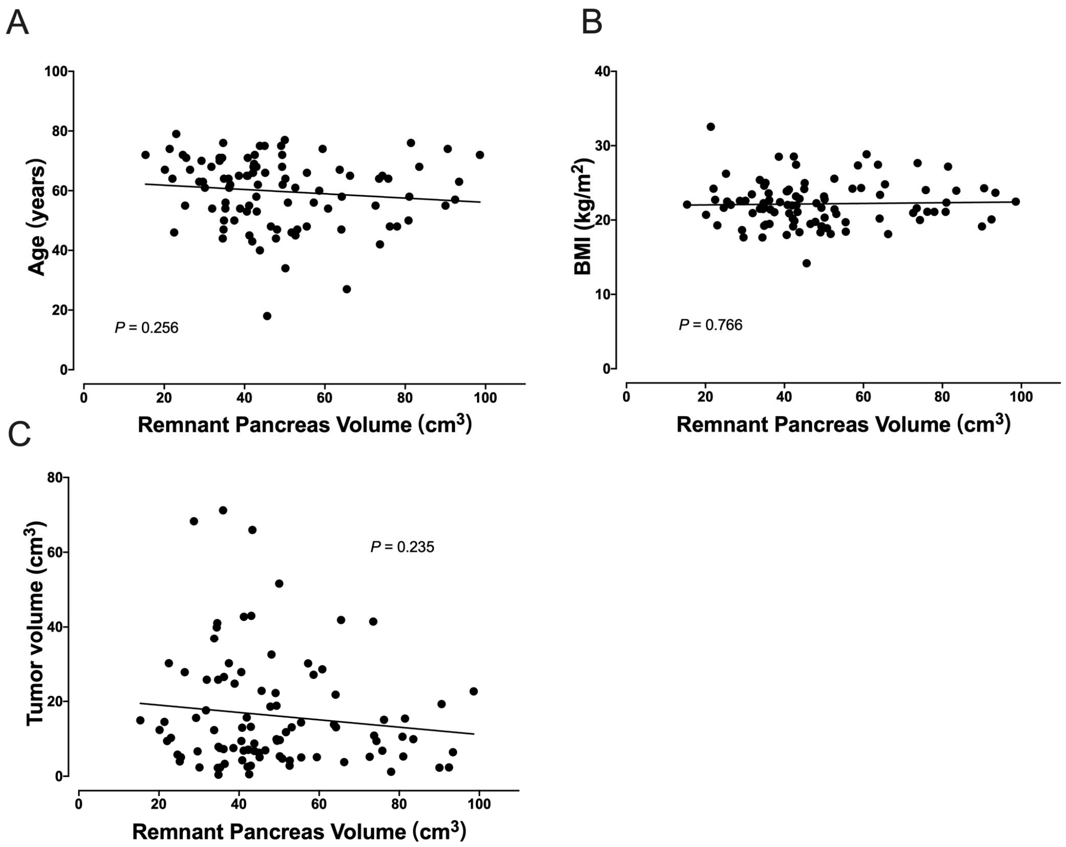

Figure S3 Correlation between remnant pancreas volume and (A) age, (B) BMI, and (C) tumor volume in PDAC patients.
